# Supplementary material for: Convergent shifts in host-associated microbial communities across environmentally elicited phenotypes
Source: Nat Commun. 2018 Mar 5;9:952. doi: 10.1038/s41467-018-03383-w (PMC5838112; doi:10.1038/s41467-018-03383-w)
Supplement: Supplementary file 1 — Supplementary Information [file 41467_2018_3383_MOESM1_ESM.pdf]

Authors: Tyler J. Carrier and Adam M. Reitzel

Title: Convergent shifts in host-associated microbial communities across environmentally elicited phenotypes

File name: Supplementary Documentation

Description: Supplementary Figures and Supplementary Tables

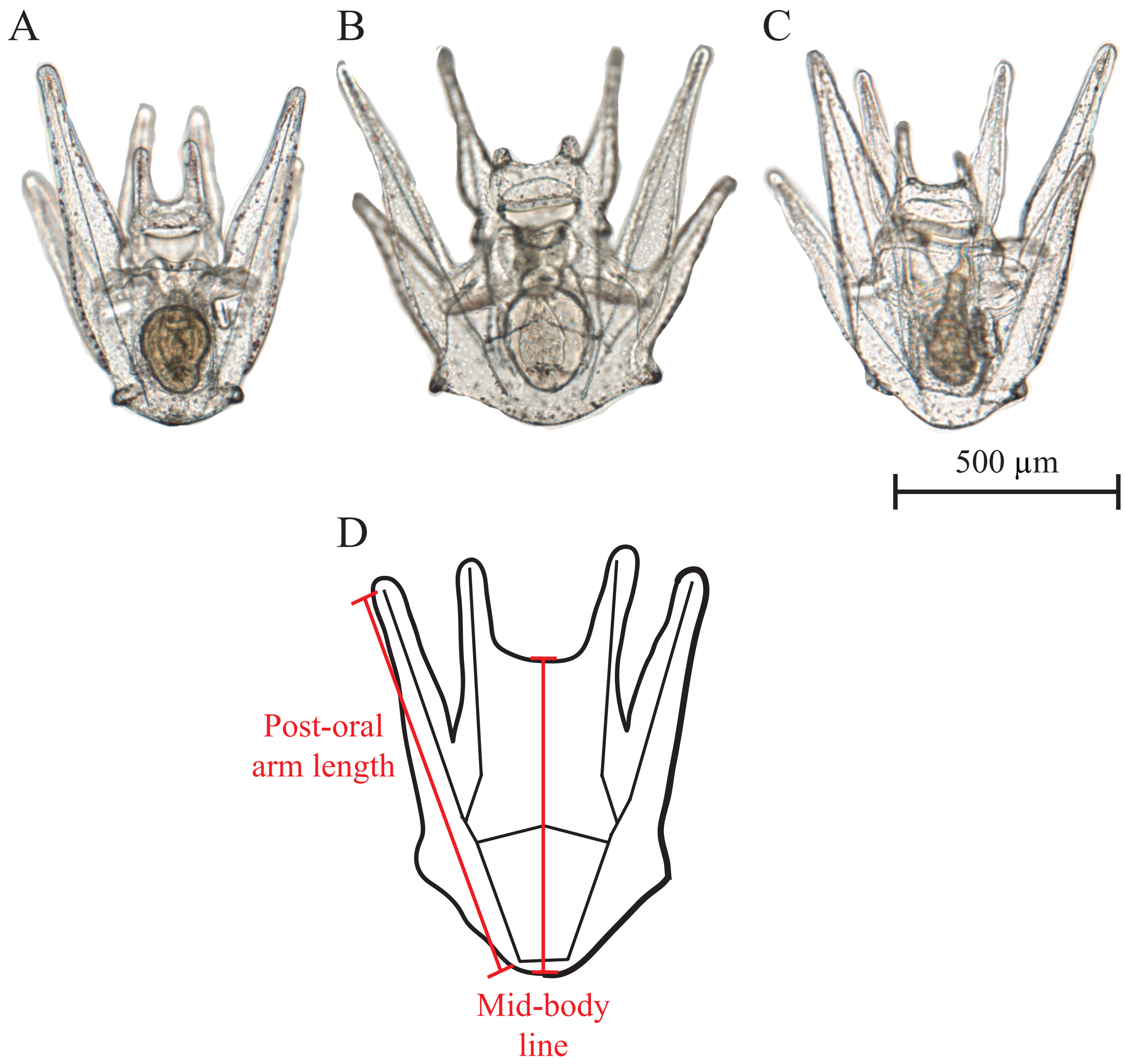

Supplementary Figure 1. Photograph of each species of echinoid larvae and representative cartoon. Microphotograph of 8-arm *Strongylocentrotus purpuratus* (A), 6-arm *Mesocentrotus franciscanus* (B), and 8-arm *S. droebachiensis* (C) larva, and (D) cartoon of 4-arm echinoid larvae with pre-drawn line for post-oral arm and mid-body line measurements.

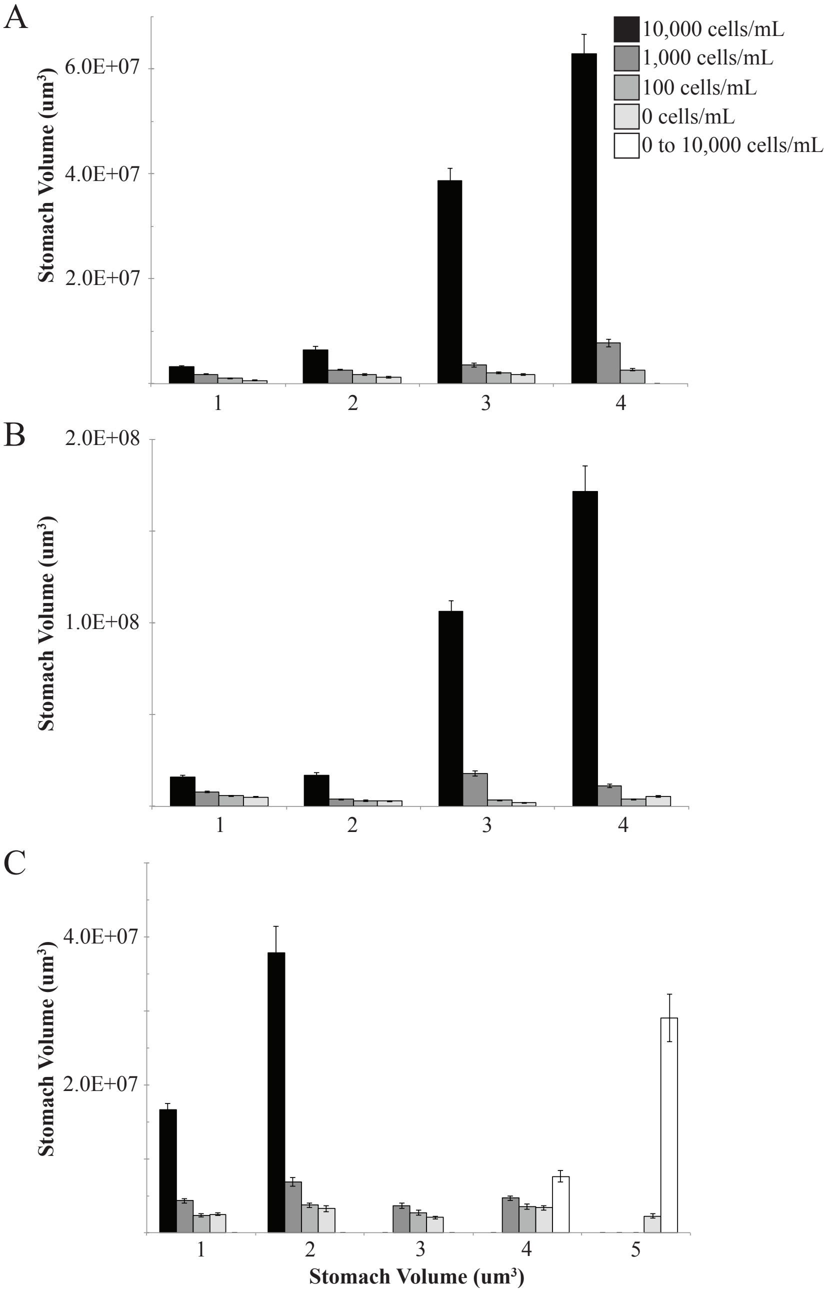

Supplementary Figure 2. Three species of echinoid larvae alter stomach volume based on feeding environment. Post-oral arm to mid body line ratio (± standard error; n=20) for *Strongylocentrotus purpuratus* (A), *Mesocentrotus franciscanus* (B), and *S. droebachiensis* (C) larvae having been fed either 10,000 (black), 1,000 (dark grey), 100 (grey), and 0 cells•mL^{-1} (light grey). For *S. droebachiensis*, larval stomach volume was also manipulated (white) by being fed 0 cells•mL^{-1} for three weeks then transferred to 10,000 cells•mL^{-1} for three weeks (*i.e.*, until metamorphosis). Data here correspond with Figure 1.

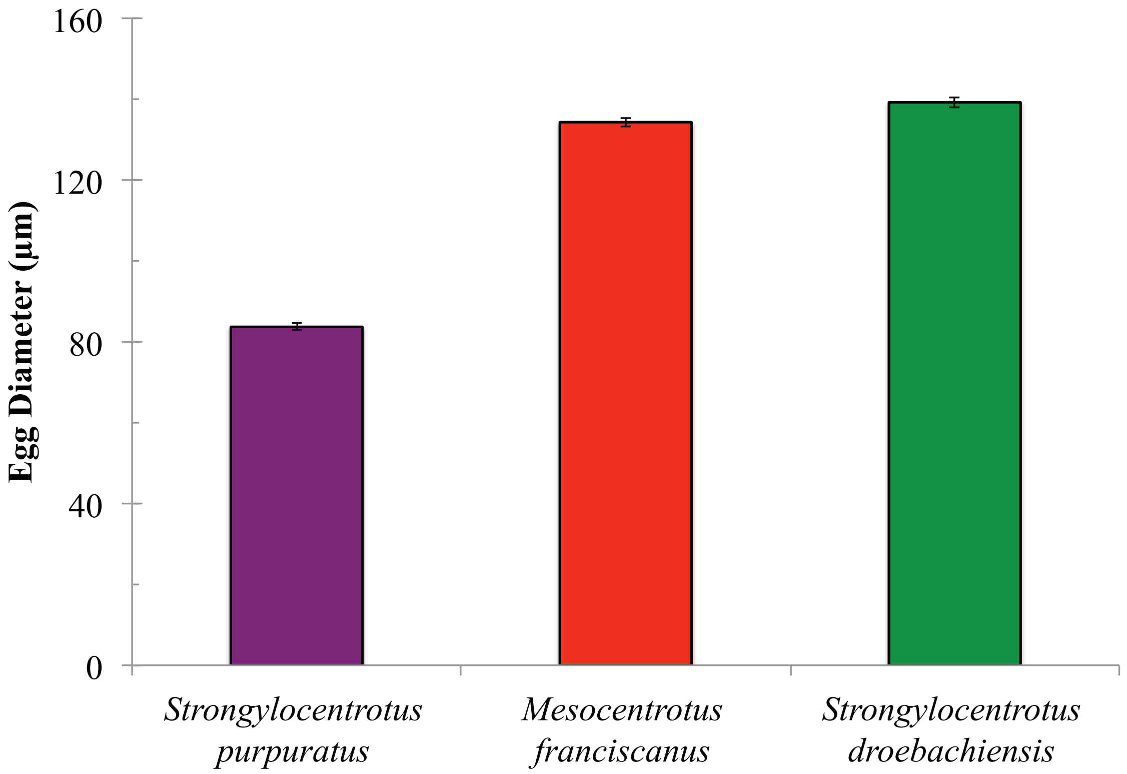

Supplementary Figure 3. Egg diameter for three species of echinoid larvae. Mean egg size (± standard error; n=20) of unfertilized eggs for *Strongylocentrotus purpuratus*, *Mesocentrotus franciscanus*, and *S. droebachiensis*.

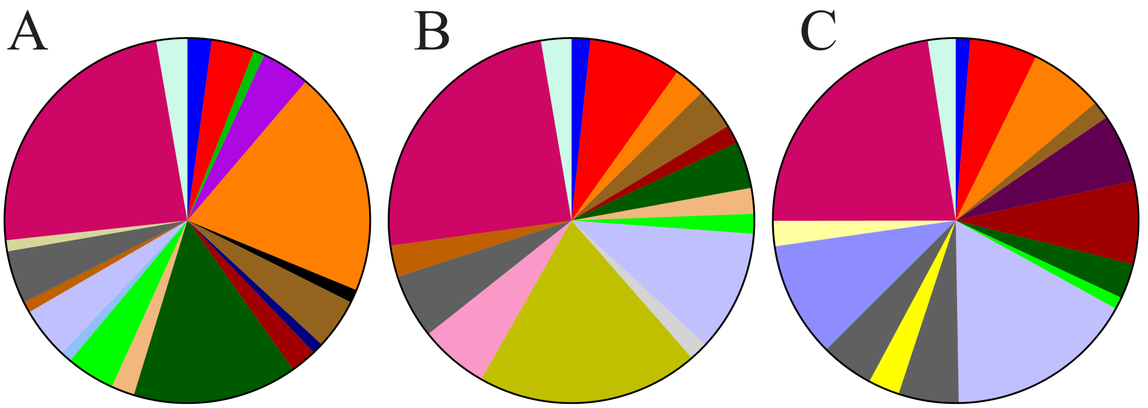

Phylum	Class	Order	Family	Genus
Bacteroidetes	Flavobacteriia	Flavobacteriales	Flavobacteriaceae	N/A
Cyanobacteria	Chloroplast	Stramenopiles	N/A	N/A
Planctomycetes	Phycisphaerae	Phycisphaerales	N/A	N/A
Proteobacteria	Alphaproteobacteria	Caulobacterales	Caulobacteraceae	N/A
		Rhizobiales	Bradyrhizobiaceae	N/A
		Rhodobacterales	Hyphomonadaceae	N/A
			Rhodobacteraceae	N/A
		Sphingomonadales	N/A	N/A
			Sphingomonadaceae	*Sphingomonas*
	Betaproteobacteria	Methylophilales	Methylophilaceae	*Methylotenera*
	Gammaproteobacteria	Alteromonadales	Alteromonadaceae	N/A
				Alteromonas
				ZD0117
				Other
			Colwelliaceae	N/A
				Colwellia
			J115	N/A
				Other
		Oceanospirillales	Oceanospirillaceae	*Oleibacter*
				Oleispira
				Other
		Pseudomonadales	Moraxellaceae	*Perlucidibaca*
			Pseudomonadaceae	*Pseudomonas*
		Xanthomonadales	Xanthomonadaceae	*Stenotrophomonas*
		Other	Other	Other
Verrucomicrobia	Verrucomicrobiae	Verrucomicrobiales	Verrucomicrobiaceae	*Persicirhabdus*
Other	Other	Other	Other	Other
Unassigned	Unassigned	Unassigned	Unassigned	Unassigned

Supplementary Figure 4. Distribution of bacterial taxa associated with *Strongylocentrotus purpuratus* larvae along the phenotypic continuum. Genera associated with *S. purpuratus* larvae pre- (A) and post-expression (B, C) of phenotypic plasticity representing at least 1% of the community, with taxa less than 1% group as 'other.' Data correspond with Figure 2A.

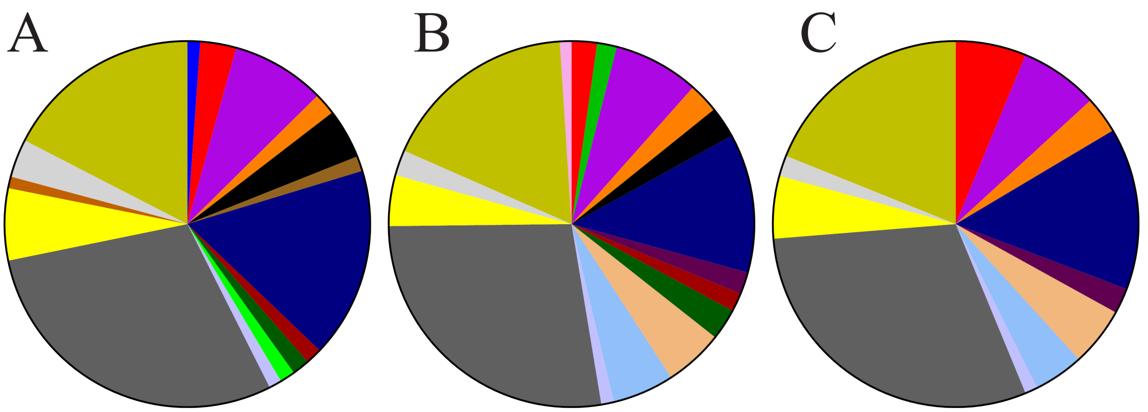

Phylum	Class	Order	Family	Genus
Bacteroidetes	Flavobacteriia	Flavobacteriales	Flavobacteriaceae	N/A
Cyanobacteria	Chloroplast	Stramenopiles	N/A	N/A
Planctomycetes	Phycisphaerae	Phycisphaerales		N/A
Proteobacteria	Alphaproteobacteria	Caulobacterales	Caulobacteraceae	N/A
		Rhizobiales	Bradyrhizobiaceae	N/A
		Rhodobacterales	Rhodobacteraceae	N/A
				Octadecabacter
		Sphingomonadales	Sphingomonadaceae	*Sphingomonas*
	Betaproteobacteria	Methylophilales	Methylophilaceae	*Methylotenera*
	Gammaproteobacteria	Alteromonadales	Alteromonadaceae	N/A
				ZD0117
			Colwelliaceae	N/A
		Oceanospirillales	Halomonadaceae	*Candidatus Portiera*
		Pseudomonadales	Moraxellaceae	*Perlucidibaca*
			Pseudomonadaceae	N/A
				Pseudomonas
		Xanthomonadales	Xanthomonadaceae	*Stenotrophomonas*
				Other
Verrucomicrobia	Verrucomicrobiae	Verrucomicrobiales	Verrucomicrobiaceae	*Rubritalea*
Other	Other	Other	Other	Other
Unassigned	Unassigned	Unassigned	Unassigned	Unassigned

Supplementary Figure 5. Distribution of bacterial taxa associated with *Mesocentrotus franciscanus* larvae along the phenotypic continuum. Genera associated with *M. franciscanus* larvae pre- (A) and post-expression (B, C) of phenotypic plasticity representing at least 1% of the community, with taxa less than 1% group as 'other.' Data correspond with Figure 2B.

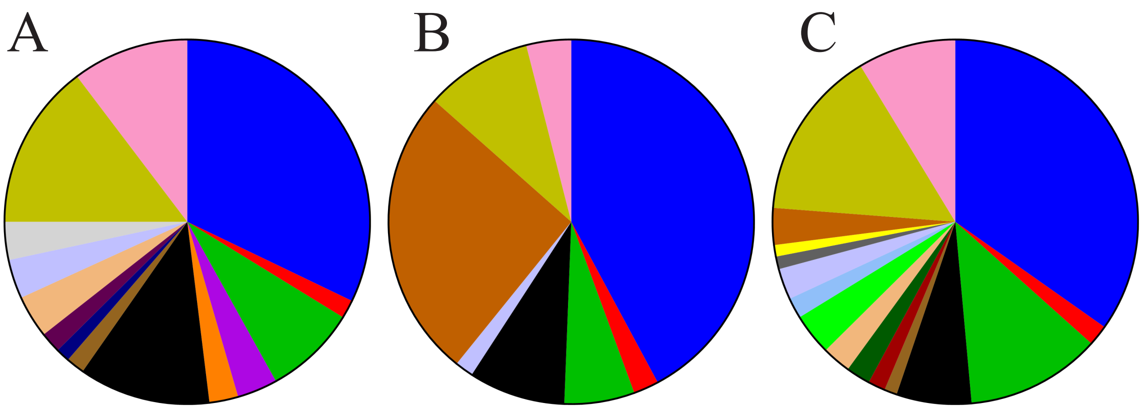

Phylum	Class	Order	Family	Genus
Bacteroidetes	Flavobacteriia	Flavobacteriales	Flavobacteriaceae	*Polaribacter*
				Other
Cyanobacteria	Chloroplast	Stramenopiles	N/A	N/A
Planctomycetes	Phycisphaerae	Phycisphaerales		N/A
	vadinHA49			N/A
Proteobacteria	Alphaproteobacteria	Rhizobiales	Bradyrhizobiaceae	N/A
		Rhodobacterales	Rhodobacteraceae	N/A
				Marivita
		Sphingomonadales	Sphingomonadaceae	*Sphingomonas*
	Betaproteobacteria	Methylophilales	Methylophilaceae	*Methylotenera*
	Gammaproteobacteria	Alteromonadales	Alteromonadaceae	N/A
				ZD0117
			Colwelliaceae	N/A
		Enterobacteriales	Enterobacteriaceae	N/A
		Oceanospirillales	Endozoicimonaceae	N/A
			Oceanospirillaceae	N/A
		Pseudomonadales	Moraxellaceae	*Perlucidibaca*
		Vibrionales	Vibrionaceae	*Vibrio*
Verrucomicrobia	Verrucomicrobiae	Verrucomicrobiales	Verrucomicrobiaceae	N/A
Other	Other	Other	Other	Other
Unassigned	Unassigned	Unassigned	Unassigned	Unassigned

Supplementary Figure 6. Distribution of bacterial taxa associated with *Strongylocentrotus droebachiensis* larvae along the phenotypic continuum. Genera associated with *S. droebachiensis* larvae pre- (A) and post-expression (B, C) of phenotypic plasticity representing at least 1% of the community, with taxa less than 1% group as 'other.' Data correspond with Figure 2C.

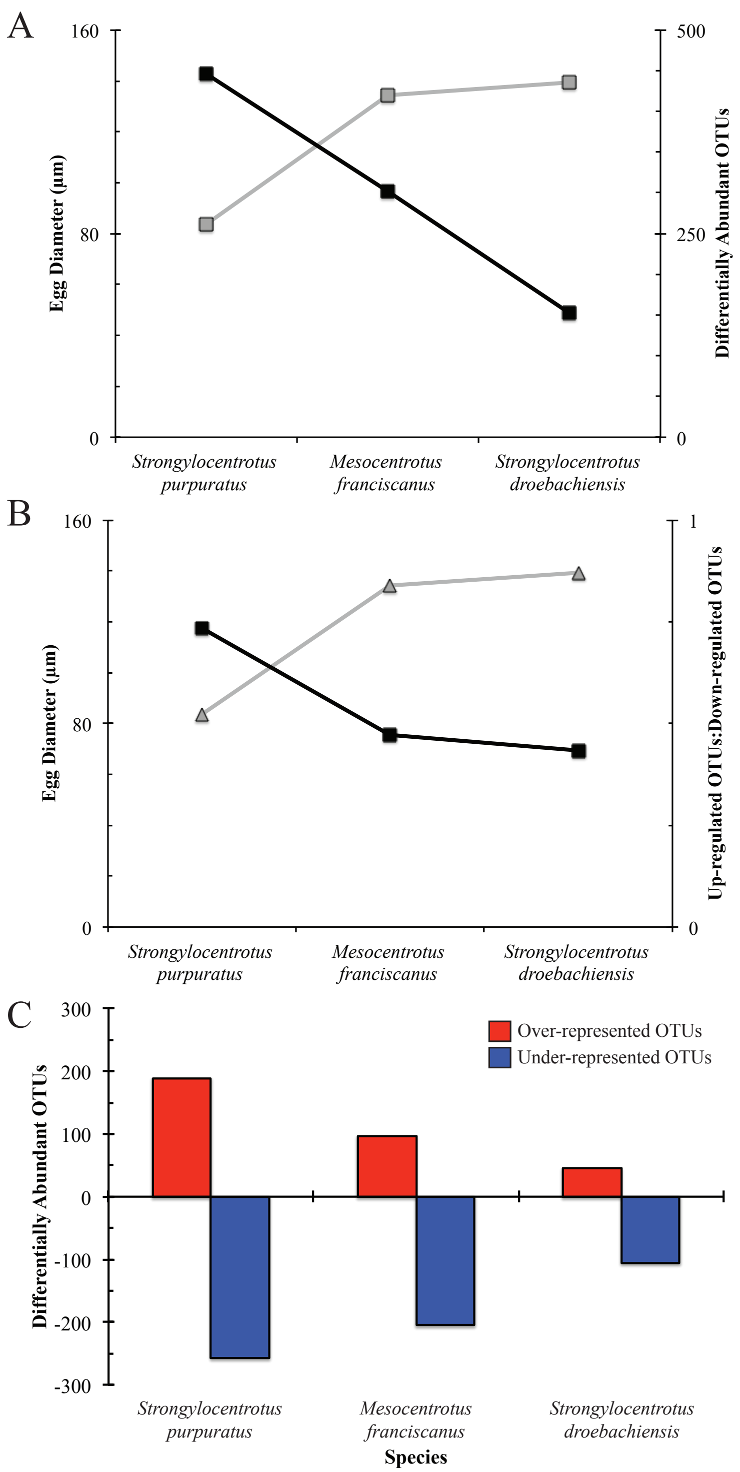

Supplementary Figure 7. Differential abundance of OTUs in relation to egg size for three species of echinoid larvae. Total (A, B) and ration of (C) over- and under-represented OTUs associated with *Strongylocentrotus purpuratus*, *Mesocentrotus franciscanus*, and *S. droebachiensis* larvae following the expression of phenotypic plasticity (black) and in relation to egg size (gray).

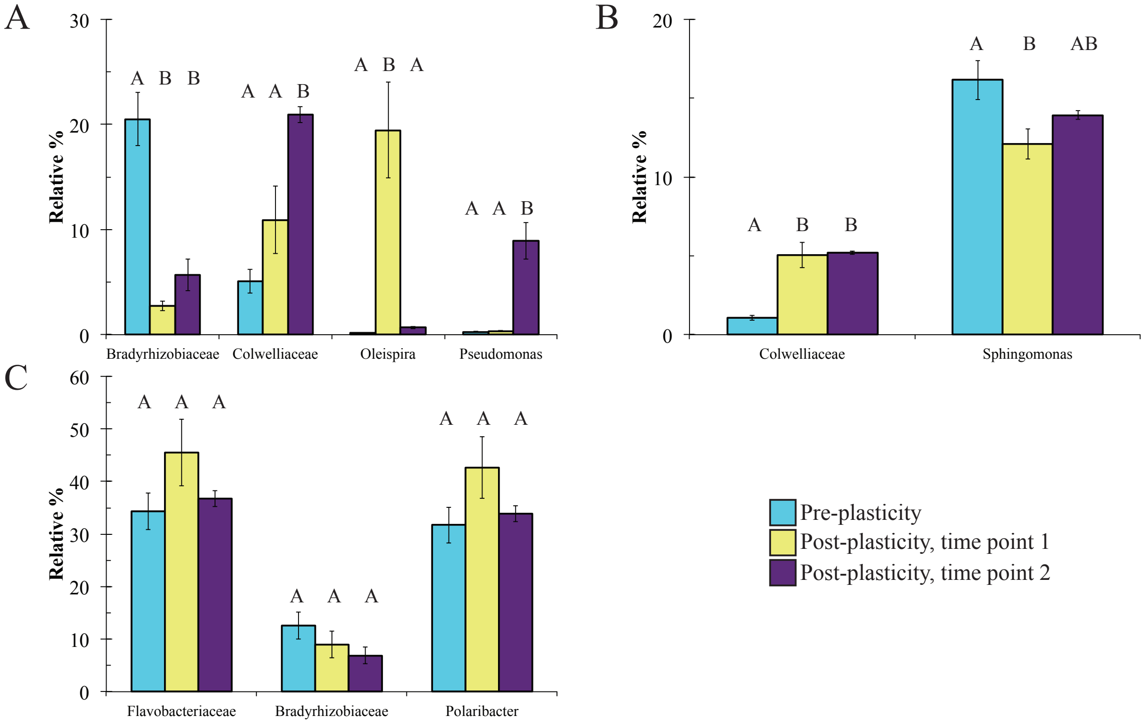

Supplementary Figure 8. Specific bacterial taxa within the proteobacteria differentially associated with larvae from three species of echinoid along the phenotypic continuum. Relative presence of specific bacterial taxa associated with *Strongylocentrotus purpuratus* (A), *Mesocentrotus franciscanus* (B), and *S. droebachiensis* (C) pre- (blue) and post-expression (yellow and purple) of phenotypic plasticity. Data correspond with Figure 2D-F.

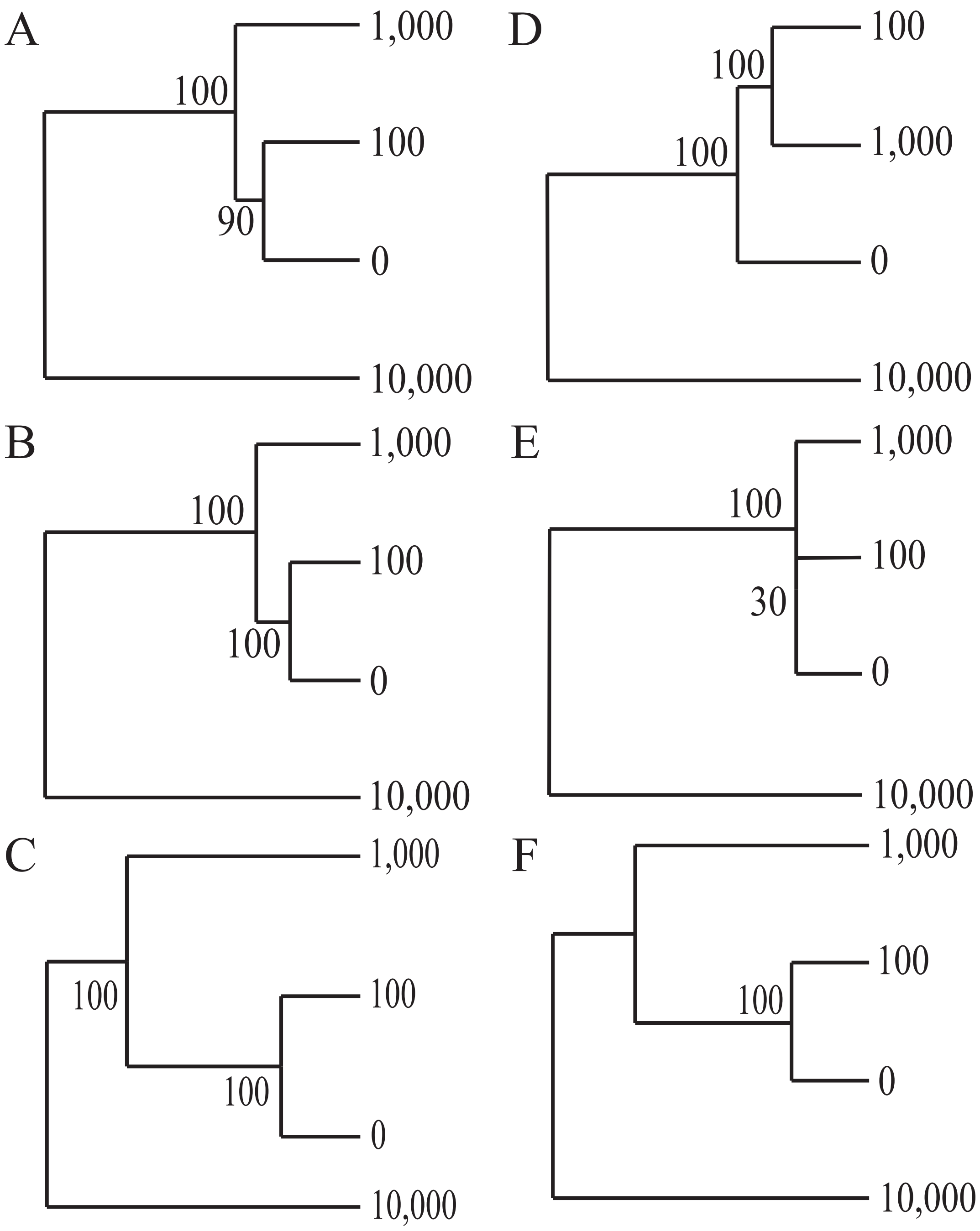

Supplementary Figure 9. Similarity between associated microbial community for three species of differentially fed echinoid larvae. Microbial dendrograms of *Strongylocentrotus purpuratus* (A, D), *Mesocentrotus franciscanus* (B, E), and *S. droebachiensis* (C, F) differentially fed at time points pre- (A-C) and post-expression (D-F) of phenotypic plasticity. Data correspond with Figure 4.

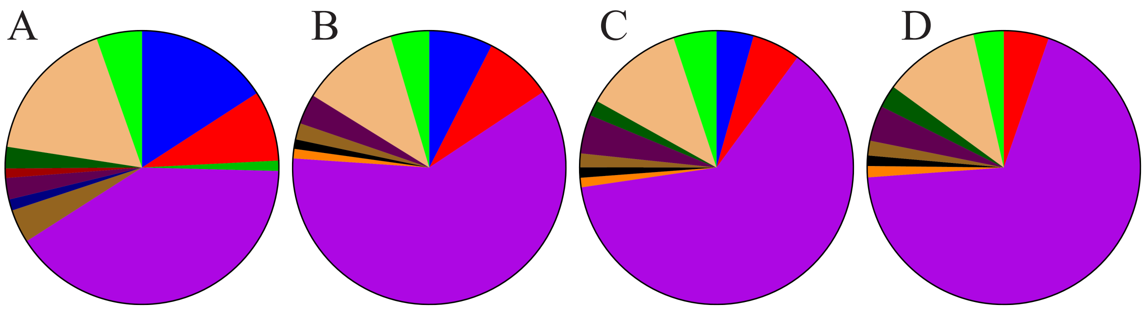

Phylum	Class	Order	Family	Genus
Bacteroidetes	Flavobacteriia	Flavobacteriales	Flavobacteriaceae	N/A
Cyanobacteria	Chloroplast	Stramenopiles	N/A	N/A
Planctomycetes	Phycisphaerae	Phycisphaerales		N/A
Proteobacteria	Alphaproteobacteria	Rhizobiales	Bradyrhizobiaceae	N/A
				Other
			Other	Other
		Rhodobacterales	Rhodobacteraceae	N/A
				Marivita
		Sphingomonadales	Sphingomonadaceae	*Sphingomonas*
	Gammaproteobacteria	Alteromonadales	Alteromonadaceae	N/A
				ZD0117
Other	Other	Other	Other	Other
Unassigned	Unassigned	Unassigned	Unassigned	Unassigned

Supplementary Figure 10. Distribution of bacterial taxa associated with differentially fed *Strongylocentrotus purpuratus* larvae pre-expression of phenotypic plasticity. Genera associated with *S. purpuratus* larvae fed either 10,000 (A), 1,000 (B), 100 (C), and 0 cells•mL^{-1} (D) following one week of differential feeding representing at least 1% of the community, with taxa less than 1% group as 'other.' Data correspond with Figure 4A.

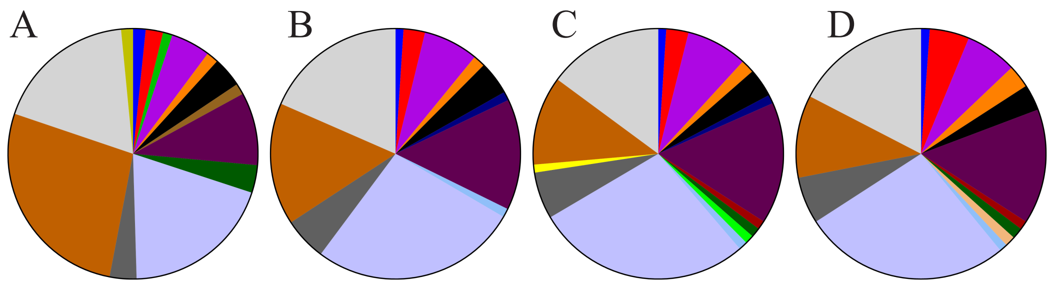

Phylum	Class	Order	Family	Genus
Bacteroidetes	Flavobacteriia	Flavobacteriales	Flavobacteriaceae	N/A
Cyanobacteria	Chloroplast	Stramenopiles	N/A	N/A
Planctomycetes	Phycisphaerae	Phycisphaerales		N/A
Proteobacteria	Alphaproteobacteria	Caulobacterales	Caulobacteraceae	N/A
		Rhizobiales	Bradyrhizobiaceae	N/A
		Rhodobacterales	Rhodobacteraceae	N/A
				Marivita
				Octadecabacter
		Sphingomonadales	Sphingomonadaceae	*Sphingomonas*
	Gammaproteobacteria	Alteromonadales	Alteromonadaceae	N/A
				ZD0117
			Colwelliaceae	N/A
		Oceanospirillales	Halomonadaceae	*Candidatus Portiera*
		Pseudomonadales	Pseudomonadaceae	N/A
				Pseudomonas
		Xanthomonadales	Xanthomonadaceae	*Stenotrophomonas*
				Other
Verrucomicrobia	Verrucomicrobiae	Verrucomicrobiales	Verrucomicrobiaceae	*Rubritalea*
Other	Other	Other	Other	Other
Unassigned	Unassigned	Unassigned	Unassigned	Unassigned

Supplementary Figure 11. Distribution of bacterial taxa associated with differentially fed *Mesocentrotus franciscanus* larvae pre-expression of phenotypic plasticity. Genera associated with *M. franciscanus* larvae fed either 10,000 (A), 1,000 (B), 100 (C), and 0 cells•mL^{-1} (D) following one week of differential feeding representing at least 1% of the community, with taxa less than 1% group as 'other.' Data correspond with Figure 4B.

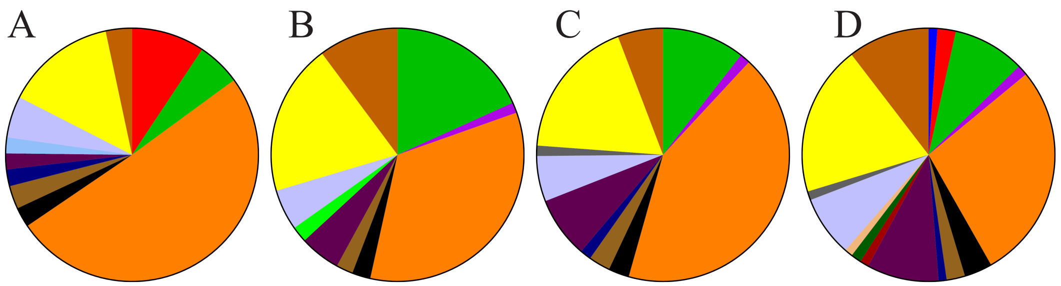

Phylum	Class	Order	Family	Genus
Bacteroidetes	Flavobacteriia	Flavobacteriales	Flavobacteriaceae	N/A
				Polaribacter
Cyanobacteria	Chloroplast	Stramenopiles	N/A	N/A
Planctomycetes	Planctomycetia	Pirellulales	Pirellulaceae	N/A
Proteobacteria	Alphaproteobacteria	Rhizobiales	Bradyrhizobiaceae	N/A
		Rhodobacterales	Rhodobacteraceae	N/A
		Sphingomonadales	Sphingomonadaceae	*Sphingomonas*
	Betaproteobacteria	Burkholderiales	Oxalobacteraceae	*Ralstonia*
	Gammaproteobacteria	Alteromonadales	Alteromonadaceae	ZD0117
		Enterobacteriales	Enterobacteriaceae	N/A
		Oceanospirillales	Halomonadaceae	*Candidatus Portiera*
			Oceanospirillaceae	N/A
		Pseudomonadales	Pseudomonadaceae	N/A
		Vibrionales	Vibrionaceae	*Vibrio*
Verrucomicrobia	Verrucomicrobiae	Verrucomicrobiales	Verrucomicrobiaceae	N/A
Verrucomicrobia				*Persicirhabdus*
Other	Other	Other	Other	Other
Unassigned	Unassigned	Unassigned	Unassigned	Unassigned

Supplementary Figure 12. Distribution of bacterial taxa associated with differentially fed *Strongylocentrotus droebachiensis* larvae pre-expression of phenotypic plasticity. Genera associated with *S. droebachiensis* larvae fed either 10,000 (A), 1,000 (B), 100 (C), and 0 cells•mL^{-1} (D) following one week of differential feeding representing at least 1% of the community, with taxa less than 1% group as 'other.' Data correspond with Figure 4C.

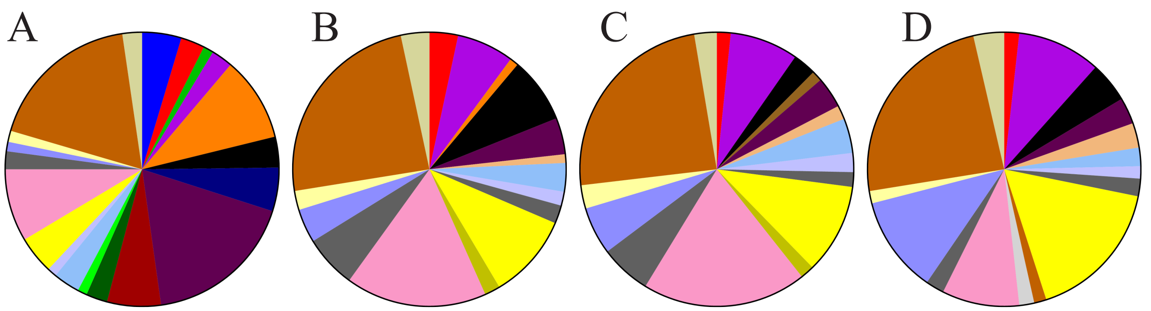

Phylum	Class	Order	Family	Genus
Bacteroidetes	[Rhodothermi]	[Rhodothermales]	[Balneolaceae]	*Balneola*
	Flavobacteriia	Flavobacteriales	Flavobacteriaceae	N/A
				Polaribacter
Cyanobacteria	Chloroplast	Stramenopiles	N/A	N/A
Planctomycetes	Phycisphaerae	Phycisphaerales		N/A
Proteobacteria	Alphaproteobacteria	Rhizobiales	Bradyrhizobiaceae	N/A
		Rhodobacterales	Hyphomonadaceae	N/A
				Oceanicaulis
			Rhodobacteraceae	N/A
				Marivita
		Other	Other	Other
	Betaproteobacteria	Methylophilales	Methylophilaceae	*Methylotenera*
	Gammaproteobacteria	N/A	N/A	N/A
		Alteromonadales	Alteromonadaceae	N/A
				Alteromonas
				ZD0117
			Colwelliaceae	N/A
			J115	N/A
				Other
		Oceanospirillales	Oceanospirillaceae	*Oleibacter*
				Oleispira
				Other
		Pseudomonadales	Moraxellaceae	*Perlucidibaca*
		Other	Other	Other
Other	Other			Other
Unassigned	Unassigned	Unassigned	Unassigned	Unassigned

Supplementary Figure 13. Distribution of bacterial taxa associated with differentially fed *Strongylocentrotus purpuratus* larvae post-expression of phenotypic plasticity. Genera associated with *S. purpuratus* larvae fed either 10,000 (A), 1,000 (B), 100 (C), and 0 cells•mL^{-1} (D) following one week of differential feeding representing at least 1% of the community, with taxa less than 1% group as ‘other.’ Data correspond with Figure 4D.

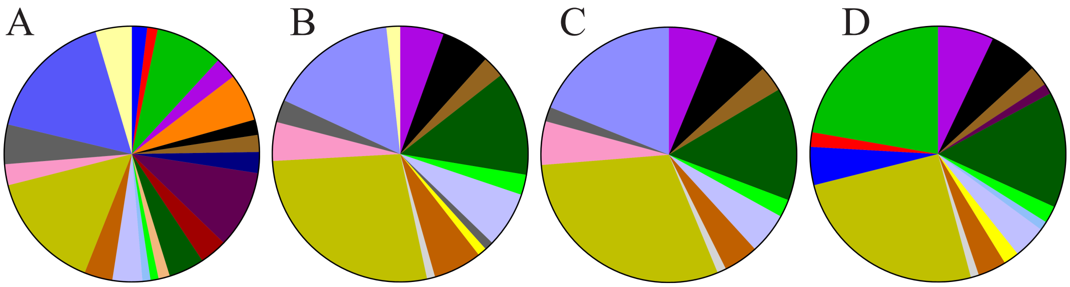

Phylum	Class	Order	Family	Genus
Bacteroidetes	[Rhodothermi]	[Rhodothermales]	[Balneolaceae]	*Balneola*
	Flavobacteriia	Flavobacteriales	Flavobacteriaceae	N/A
				Polaribacter
Cyanobacteria	Chloroplast	Stramenopiles	N/A	N/A
Planctomycetes	Phycisphaerae	Phycisphaerales		N/A
Proteobacteria	Alphaproteobacteria	Caulobacterales	Caulobacteraceae	N/A
		Rhizobiales	Bradyrhizobiaceae	N/A
		Rhodobacterales	Hyphomonadaceae	*Oceanicaulis*
			Rhodobacteraceae	N/A
				Marivita
		Sphingomonadales	Sphingomonadaceae	*Sphingomonas*
		Other	Other	Other
	Betaproteobacteria	Methylophilales	Methylophilaceae	*Methylotenera*
	Gammaproteobacteria	Alteromonadales	Alteromonadaceae	N/A
			Colwelliaceae	N/A
			J115	N/A
		Oceanospirillales	Oceanospirillaceae	*Oleispira*
		Pseudomonadales	Moraxellaceae	*Perlucidibaca*
			Pseudomonadaceae	N/A
				Pseudomonas
		Xanthomonadales	Xanthomonadaceae	*Stenotrophomonas*
Verrucomicrobia	Verrucomicrobiae	Verrucomicrobiales	Verrucomicrobiaceae	*Rubritalea*
Other	Other	Other	Other	Other
Unassigned	Unassigned	Unassigned	Unassigned	Unassigned

Supplementary Figure 14. Distribution of bacterial taxa associated with differentially fed *Mesocentrotus franciscanus* larvae post-expression of phenotypic plasticity. Genera associated with *M. franciscanus* larvae fed either 10,000 (A), 1,000 (B), 100 (C), and 0 cells•mL^{-1} (D) following one week of differential feeding representing at least 1% of the community, with taxa less than 1% group as 'other.' Data correspond with Figure 4E.

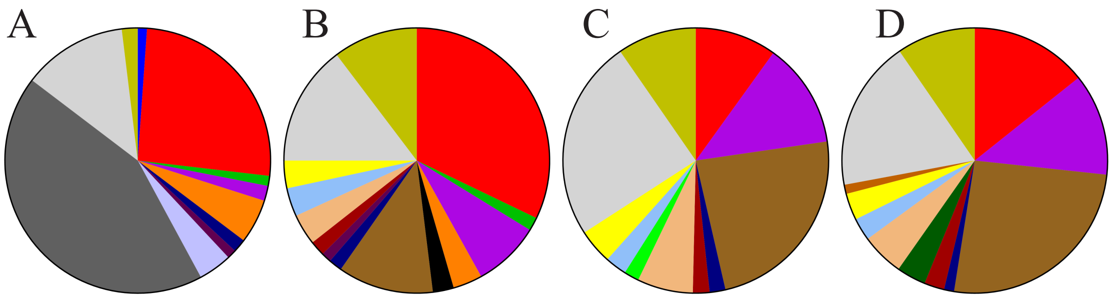

Phylum	Class	Order	Family	Genus
Bacteroidetes	[Saprospirae]	[Saprospirales]	Saprospiraceae	*Saprospira*
	Flavobacteriia	Flavobacteriales	Flavobacteriaceae	*Polaribacter*
				Other
Cyanobacteria	Chloroplast	Stramenopiles	N/A	N/A
Planctomycetes	Phycisphaerae	Phycisphaerales		N/A
Planctomycetes	vadinHA49	N/A		N/A
Proteobacteria	Alphaproteobacteria	Rhizobiales	Bradyrhizobiaceae	N/A
		Rhodobacterales	Rhodobacteraceae	N/A
				Marivita
		Sphingomonadales	Sphingomonadaceae	*Sphingomonas*
	Gammaproteobacteria	Alteromonadales	Alteromonadaceae	N/A
				ZD0117
			Shewanellaceae	*Shewanella*
		Oceanospirillales	Endozoicimonaceae	N/A
		Vibrionales	Vibrionaceae	N/A
				Vibrio
Verrucomicrobia	Verrucomicrobiae	Verrucomicrobiales	Verrucomicrobiaceae	N/A
				Persicirhabdus
Other	Other	Other	Other	Other
Unassigned	Unassigned	Unassigned	Unassigned	Unassigned

Supplementary Figure 15. Distribution of bacterial taxa associated with differentially fed *Strongylocentrotus droebachiensis* larvae post-expression of phenotypic plasticity. Genera associated with *S. droebachiensis* larvae fed either 10,000 (A), 1,000 (B), 100 (C), and 0 cells•mL^{-1} (D) following one week of differential feeding representing at least 1% of the community, with taxa less than 1% group as 'other.' Data correspond with Figure 4F.

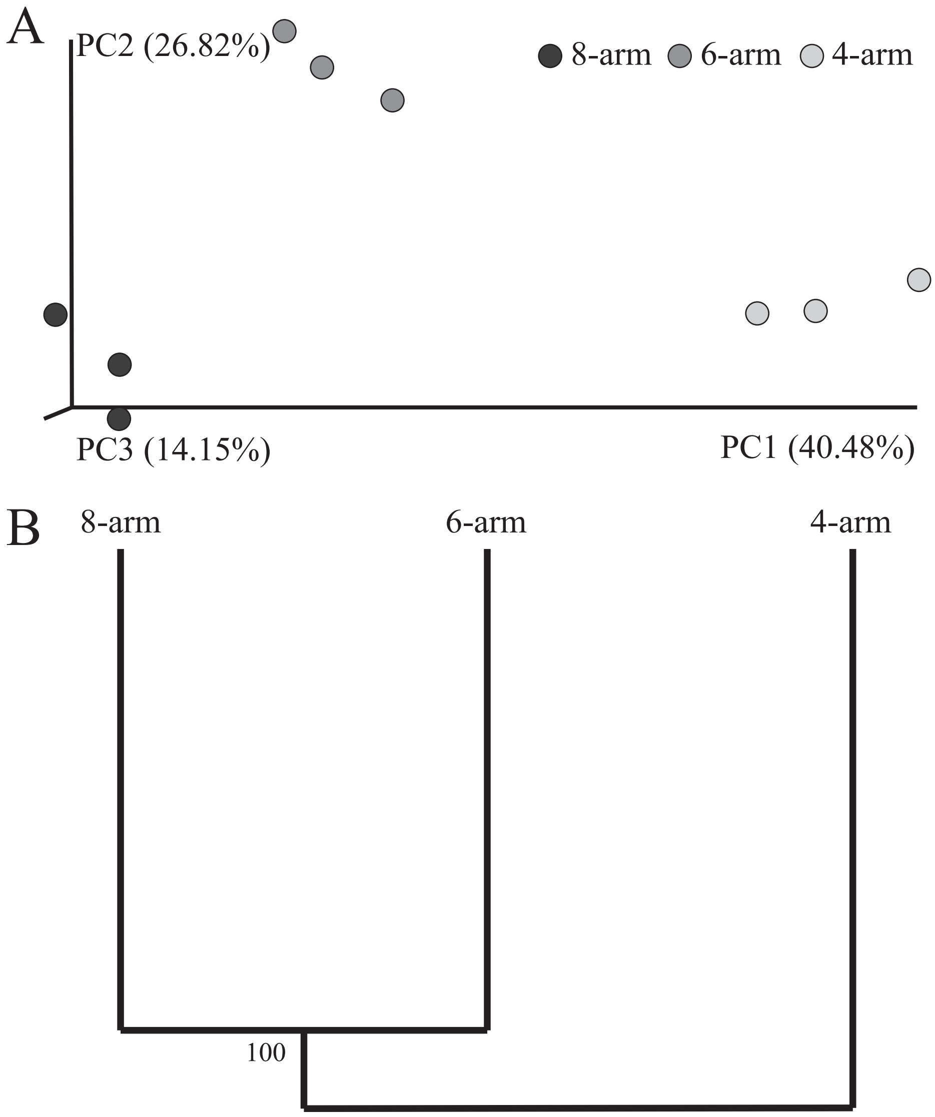

Supplementary Figure 16. Developmental stage-specific microbial community for *Strongylocentrotus purpuratus* larvae. Community similarity of the associated microbiota for *Strongylocentrotus purpuratus* larvae at the 4- (light grey), 6- (grey), and 8-arm (black) stages (A), with respective microbial dendrogram (B).

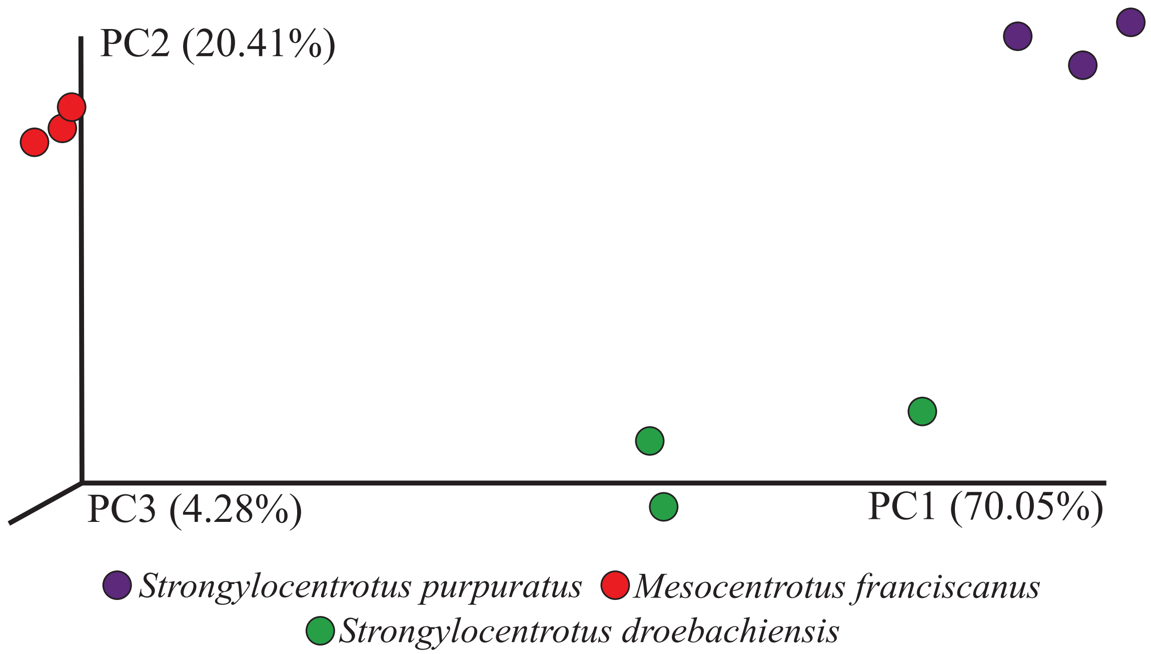

Supplementary Figure 17. Species-specific associated microbial communities for three species of echinoid larvae. Community similarity of the associated microbiota for *Strongylocentrotus purpuratus* (purple), *Mesocentrotus franciscanus* (red), and *S. droebachiensis* (green) prior to feeding.

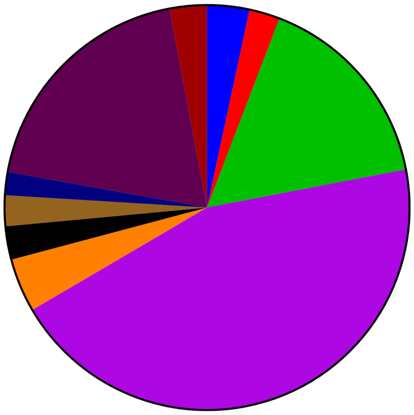

Phylum	Class	Order	Family	Genus
Bacteroidetes	Flavobacteriia	Flavobacteriales	Flavobacteriaceae	N/A
				Polaribacter
Cyanobacteria	Chloroplast	Stramenopiles	N/A	N/A
Proteobacteria	Alphaproteobacteria	Rhizobiales	Bradyrhizobiaceae	N/A
		Rhodobacterales	Rhodobacteraceae	N/A
		Sphingomonadales	Sphingomonadaceae	*Sphingomonas*
	Gammaproteobacteria	Alteromonadales	Alteromonadaceae	N/A
			Colwelliaceae	N/A
Other	Other	Other	Other	Other
Unassigned	Unassigned	Unassigned	Unassigned	Unassigned

Supplementary Figure 18. Bacterial taxa associated with prefeeding *Strongylocentrotus purpuratus* larvae. Genera associated with *S. purpuratus* larvae prior to feeding representing at least 1% of the community, with taxa less than 1% group as 'other.' Data correspond with Supplementary Figure 18.

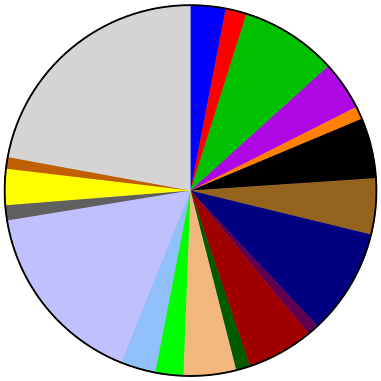

Phylum	Class	Order	Family	Genus
Bacteroidetes	[Saprospirae]	[Saprospirales]	Saprospiraceae	N/A
	Flavobacteriia	Flavobacteriales	Cryomorphaceae	*Crocinitomix*
			Flavobacteriaceae	N/A
				Polaribacter
Cyanobacteria	Chloroplast	Stramenopiles	N/A	N/A
Proteobacteria	Alphaproteobacteria	Caulobacterales	Caulobacteraceae	N/A
		Rhodobacterales	Rhodobacteraceae	N/A
		Sphingomonadales	Sphingomonadaceae	*Sphingomonas*
	Gammaproteobacteria	Alteromonadales	N/A	N/A
			Alteromonadaceae	N/A
				Alteromonas
			Colwelliaceae	N/A
		Oceanospirillales	Oceanospirillaceae	*Marinomonas*
				Oleispira
		Pseudomonadales	Pseudomonadaceae	*Pseudomonas*
		Vibrionales	Pseudoalteromonadaceae	*Pseudoalteromonas*
		Xanthomonadales	Xanthomonadaceae	*Stenotrophomonas*
		Other	Other	Other
Other	Other			Other

Supplementary Figure 19. Bacterial taxa associated with prefeeding *Mesocentrotus franciscanus* larvae. Genera associated with *M. franciscanus* larvae prior to feeding representing at least 1% of the community, with taxa less than 1% group as 'other.' Data correspond with Supplementary Figure 18.

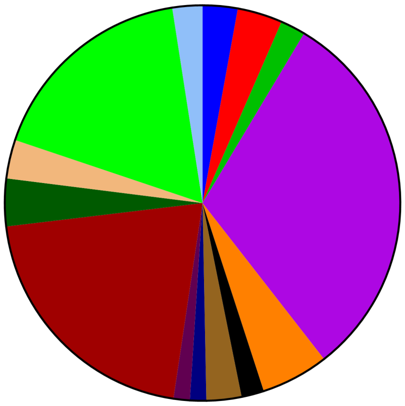

**Phylum**	**Class**	**Order**	**Family**	**Genus**
Bacteroidetes	Flavobacteriia	Flavobacteriales	Flavobacteriaceae	N/A
				Polaribacter
Cyanobacteria	Chloroplast	Stramenopiles	N/A	N/A
Proteobacteria	Alphaproteobacteria	Rhizobiales	Bradyrhizobiaceae	N/A
		Rhodobacterales	Rhodobacteraceae	N/A
		Sphingomonadales	Sphingomonadaceae	*Sphingomonas*
	Gammaproteobacteria	Alteromonadales	Alteromonadaceae	N/A
			Colwelliaceae	N/A
		Oceanospirillales	Oceanospirillaceae	N/A
				Marinomonas
		Vibrionales	Vibrionaceae	N/A
				Vibrio
Other	Other	Other	Other	Other
Unassigned	Unassigned	Unassigned	Unassigned	Unassigned

Supplementary Figure 20. Bacterial taxa associated with prefeeding *Strongylocentrotus droebachiensis* larvae. Genera associated with *S. droebachiensis* larvae prior to feeding representing at least 1% of the community, with taxa less than 1% group as 'other.' Data correspond with Supplementary Figure 18.

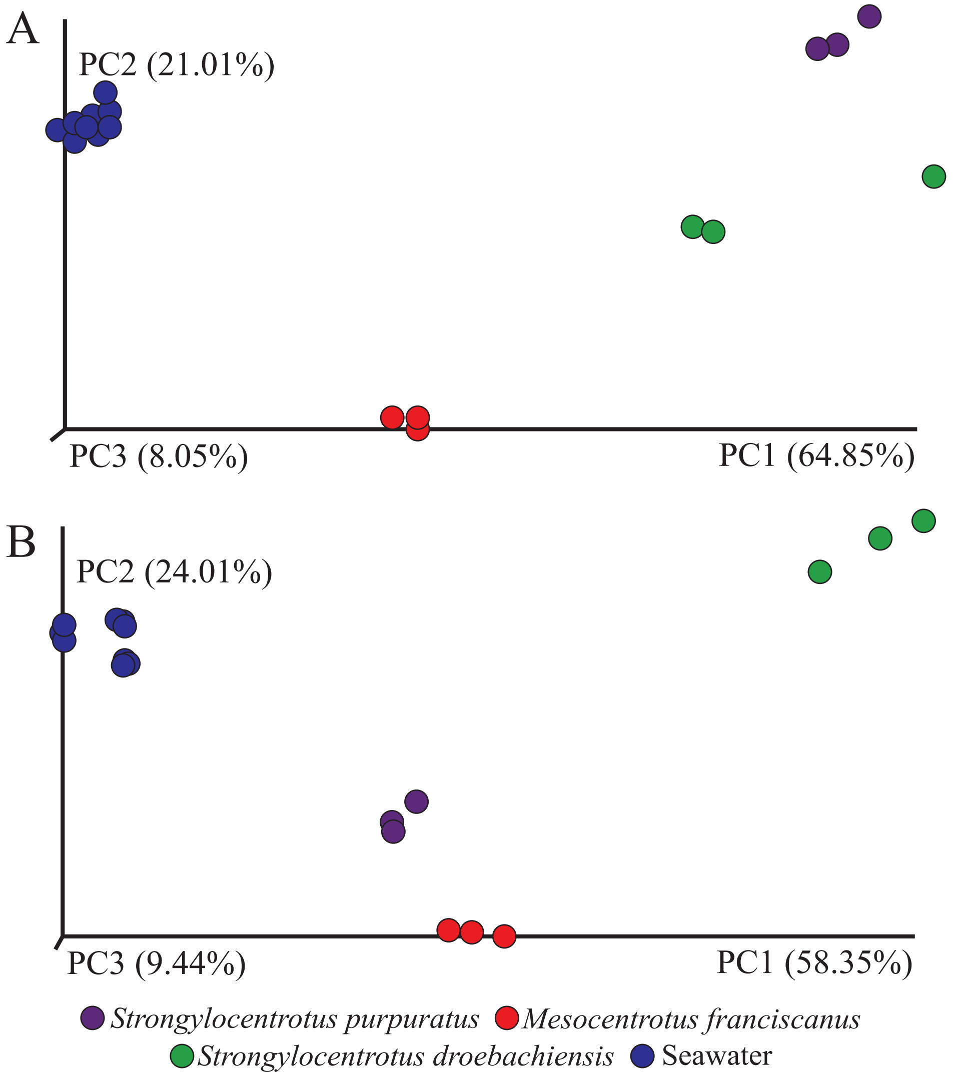

Supplementary Figure 21. Similarity of the associated microbial community for three species of echinoids at two larval stages. Community similarity of the associated microbiota for *Strongylocentrotus purpuratus* (purple), *Mesocentrotus franciscanus* (red), and *S. droebachiensis* (green) and seawater (blue) pre- (A) and post-feeding (B).

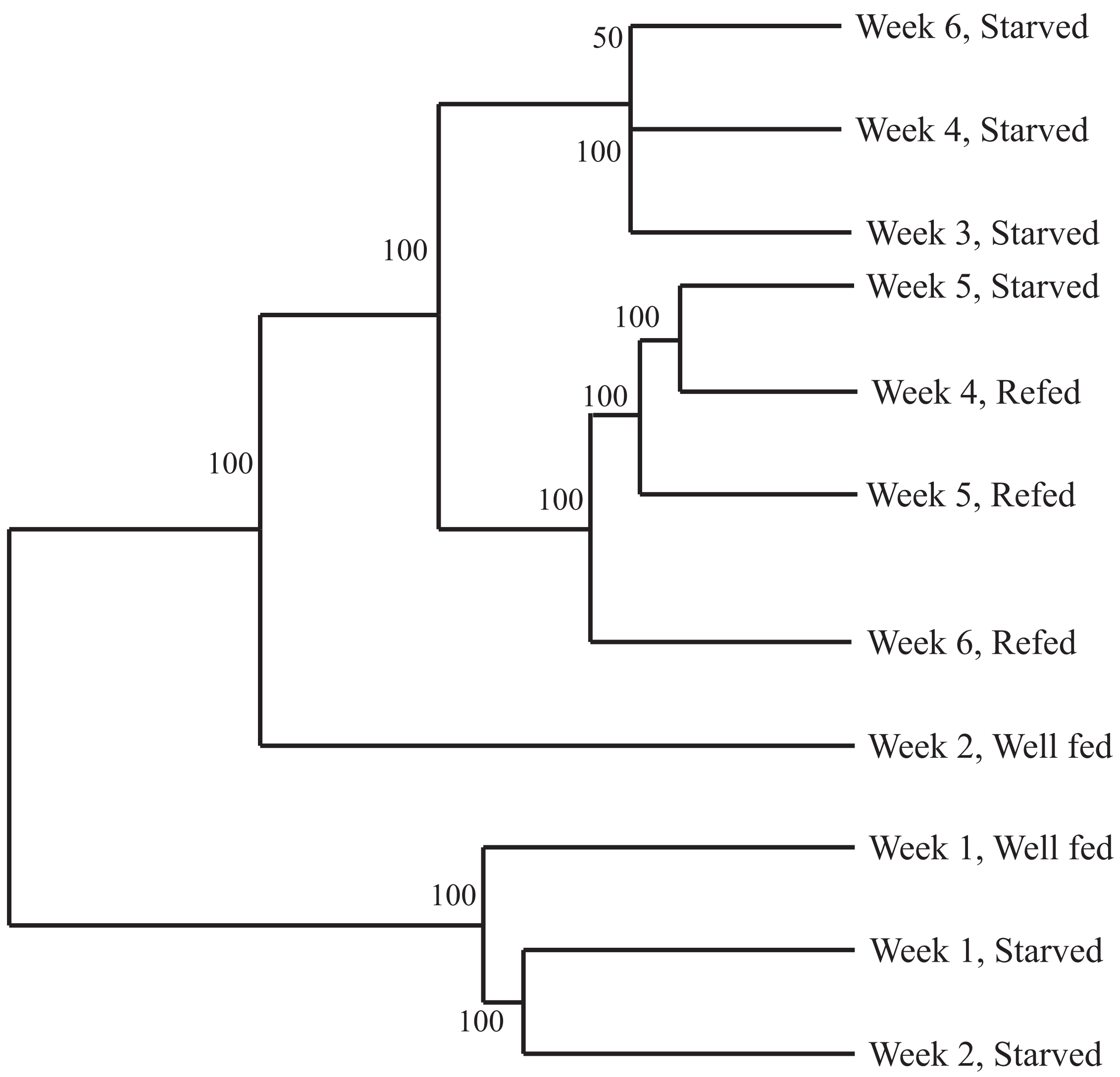

Supplementary Figure 22. Similarity between associated microbial communities for dietarily manipulated larvae. Microbial dendrograms of *Strongylocentrotus droebachiensis* larvae fed 10,000 cells•mL^{-1} until metamorphosis and 0 cells•mL^{-1} for three weeks, versus larvae fed 0 cells•mL^{-1} for three weeks then switched to 10,000 cells•mL^{-1} until metamorphosis. Data correspond with Figure 6.

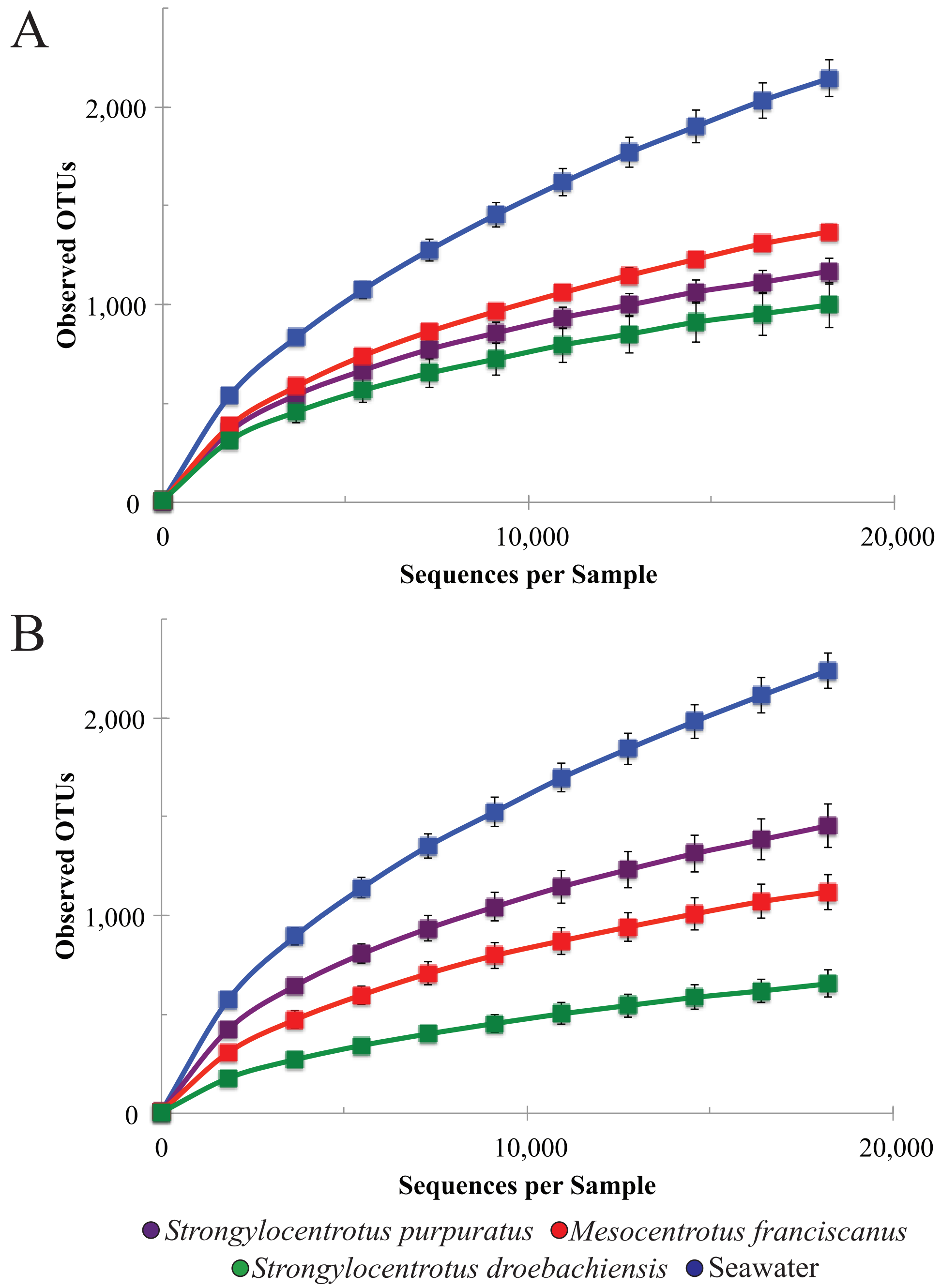

Supplementary Figure 23. Alpha rarefaction curves for three species of echinoids at two larval stages and of the seawater. Alpha rarefaction curves for *Strongylocentrotus purpuratus* (purple), *Mesocentrotus franciscanus* (red), and *S. droebachiensis* (green) and seawater (blue) pre- (A) and post-feeding (B) based on rarefaction depth (*i.e.*, 18,225) used for all PCoA plots.

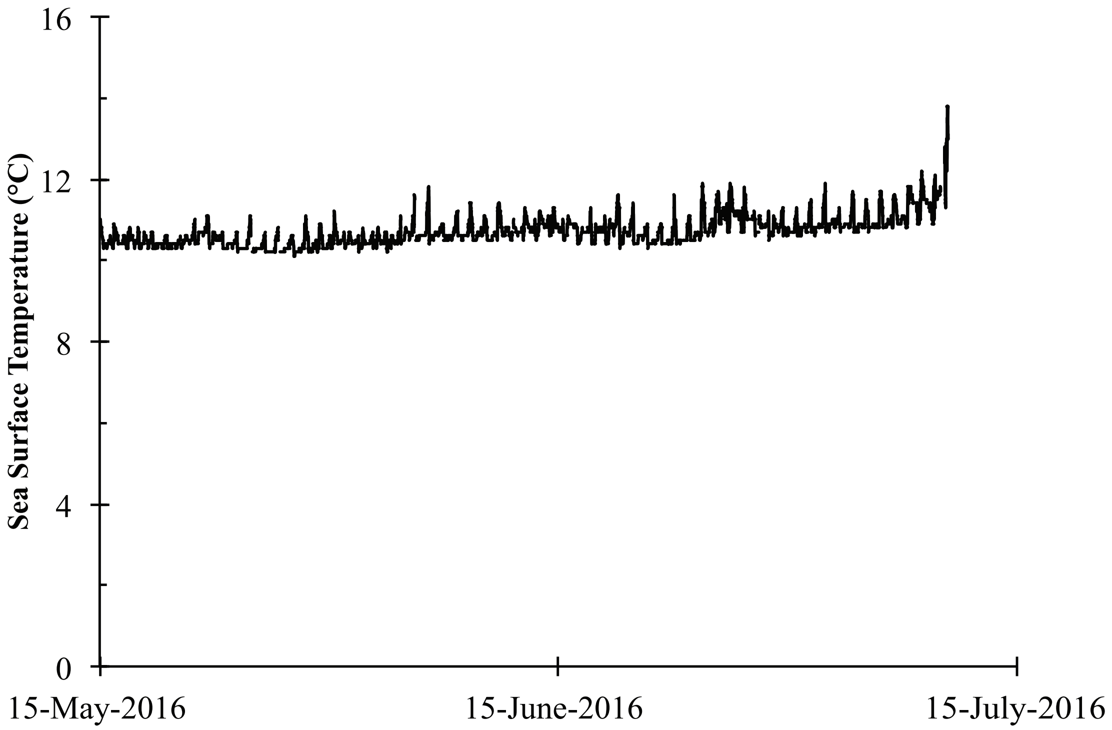

Supplementary Figure 24. *in situ* measurements of sea surface temperature at Friday Harbor, WA. Sea surface temperature at Friday Harbor, WA (NOAA station, FRDW1; 48°32'43" N 123°0'44" W) for the entirety of larval experimentation (15 May 2016 – 15 July 2016).

Supplementary Table 1. Larval developmental stages across dietary states ('--' designating no sample)

Strongylocentrotus purpuratus	**Week 1**	**Week 2**	**Week 3**	**Week 4**
**Diet = 10,000**	4-arm	4-, 6-arm	8-arm	8-arm
**Diet = 1,000**	4-arm	4-arm	6-arm	6-, 8-arm
**Diet = 100**	4-arm	4-arm	4-arm	4-arm
**Diet = 0**	4-arm	4-arm	4-arm	--

Mesocentrotus franciscanus	**Week 1**	**Week 2**	**Week 3**	**Week 4**
**Diet = 10,000**	4-arm	8-arm	8-arm	8-arm
**Diet = 1,000**	4-arm	6-arm	6-arm	6-arm
**Diet = 100**	4-arm	4-arm	4-arm	4-arm
**Diet = 0**	4-arm	4-arm	4-arm	4-arm

Strongylocentrotus droebachiensis	**Week 1**	**Week 2**	**Week 3**	**Week 4**	**Week 5**
**Diet = 10,000**	6-arm	8-arm	--	--	--
**Diet = 1,000**	6-arm	6-arm	6-arm	6-arm	--
**Diet = 100**	4-arm	4-arm	4-arm	4-arm	--
**Diet = 0**	4-arm	4-arm	4-arm	4-arm	4-arm
**Diet = 0 to 10,000**	--	--	--	6-arm	6-, 8-arm

Supplementary Table 2. Two-way ANOVA results[1] for larval morphology

	Effect	*df*	SS	*F*-ratio	*p*
Strongylocentrotus purpuratus					
	Week	2	0.522	29.197	**<0.0001**
	Diet	2	2.962	165.562	**<0.0001**
	Week*Diet	8	1.344	18.780	**<0.0001**
	Week 1	3	0.301	11.199	**<0.0001**
	Week 2	3	0.675	25.148	**<0.0001**
	Week 3	3	1.959	73.004	**<0.0001**
	Week 4	3	1.557	58.023	**<0.0001**
	Diet = 10,000	3	0.450	16.761	**<0.0001**
	Diet = 1,000	3	0.076	2.845	**0.038**
	Diet = 100	3	0.331	12.326	**<0.0001**
	Diet = 0	3	1.092	40.714	**<0.0001**
Mesocentrotus franciscanus	Effect	*df*	SS	*F*-ratio	*p*
	Week	2	1.115	28.748	**<0.0001**
	Diet	2	9.714	250.410	**<0.0001**
	Week*Diet	8	3.627	31.168	**<0.0001**
	Week 1	3	0.178	4.586	**0.0037**
	Week 2	3	2.167	55.853	**<0.0001**
	Week 3	3	3.480	89.700	**<0.0001**
	Week 4	3	7.577	195.314	**<0.0001**
	Diet = 10,000	3	0.864	22.285	**<0.0001**
	Diet = 1,000	3	0.317	8.181	**<0.0001**
	Diet = 100	3	0.793	20.453	**<0.0001**
	Diet = 0	3	2.768	71.359	**<0.0001**
Strongylocentrotus droebachiensis	Effect	*df*	SS	*F*-ratio	*p*
	Week	1	0.923	119.776	**<0.0001**
	Diet	2	0.137	8.876	**0.0002**
	Week*Diet	7	0.591	10.956	**<0.0001**
	Week 1	3	0.104	4.506	**0.0042**
	Week 2	3	0.636	27.481	**<0.0001**
	Week 3	3	0.109	4.733	**0.0031**
	Week 4	3	0.507	21.917	**<0.0001**
	Diet = 10,000	3	0.807	34.879	**<0.0001**
	Diet = 1,000	3	0.295	12.745	**<0.0001**
	Diet = 100	3	0.166	7.198	**0.0001**
	Diet = 0	3	0.738	31.897	**<0.0001**

[1] *df*: degrees of freedom; SS: sum-of-squares; *F*-ratio: F-statistic

Supplementary Table 3. Two-way ANOVA results[2] for larval stomach volume

Strongylocentrotus purpuratus

Effect	*df*	SS	*F*-ratio	*p*
Week	2	4.5E+15	84.754	**<0.0001**
Diet	2	3.3E+16	622.449	**<0.0001**
Week*Diet	8	3.0E+16	140.378	**<0.0001**
Week 1	3	4.0E+14	4.653	**0.003**
Week 2	3	3.0E+13	0.335	0.800
Week 3	3	4.0E+14	5.198	**0.002**
Week 4	3	5.0E+16	601.434	**<0.0001**
Diet = 10,000	3	8.0E+13	1.002	0.392
Diet = 1,000	3	3.0E+14	4.334	**0.005**
Diet = 100	3	2.0E+16	247.235	**<0.0001**
Diet = 0	3	5.0E+16	574.317	**<0.0001**

Mesocentrotus franciscanus

Effect	*df*	SS	F-ratio	*p*
Week	3	9.4E+16	103.686	**<0.0001**
Diet	3	3.1E+17	343.181	**<0.0001**
Week*Diet	9	2.5E+17	92.261	**<0.0001**
Week 1	3	2.0E+14	0.188	0.9045
Week 2	3	9.0E+13	0.095	0.9626
Week 3	3	2.0E+15	2.316	0.0758
Week 4	3	3.0E+17	377.871	**<0.0001**
Diet = 10,000	3	2.0E+15	1.657	0.1763
Diet = 1,000	3	3.0E+15	3.187	**0.0241**
Diet = 100	3	1.0E+17	164.025	**<0.0001**
Diet = 0	3	4.0E+17	451.096	**<0.0001**

Strongylocentrotus droebachiensis

Effect	*df*	SS	*F*-ratio	*p*
Week	1	1.7E+15	76.456	**<0.0001**
Diet	2	2.0E+14	4.618	**0.0107**
Week*Diet	7	2.9E+15	19.198	**<0.0001**
Week 1	3	2.0E+13	0.344	0.7934
Week 2	3	3.0E+13	0.393	0.7585
Week 3	3	1.0E+14	1.791	0.1493
Week 4	3	5.0E+15	69.420	**<0.0001**
Diet = 10,000	3	3.0E+15	42.776	**<0.0001**
Diet = 1,000	3	2.0E+16	254.487	**<0.0001**
Diet = 100	3	1.0E+15	15.247	**<0.0001**
Diet = 0	3	6.0E+14	8.374	**<0.0001**

[2] *df*: degrees of freedom; SS: sum-of-squares; *F*-ratio: F-statistic

Supplementary Table 4. One-way ANOVA results[3] for trade-off in associating with proteobacteria across phenotypes

Strongylocentrotus purpuratus		Effect	*df*	SS	*F*-ratio	*p*
	α-proteobacteria	Phenotype	2	0.059	19.524	**0.002**
	ϒ-proteobacteria	Phenotype	2	0.070	17.271	**0.003**
Mesocentrotus franciscanus		Effect	*df*	SS	*F*-ratio	*p*
	α-proteobacteria	Phenotype	2	0.008	12.548	**0.011**
	ϒ-proteobacteria	Phenotype	2	0.007	19.194	**0.005**
Strongylocentrotus droebachiensis		Effect	*df*	SS	*F*-ratio	*p*
	α-proteobacteria	Phenotype	2	0.013	2.964	0.142
	ϒ-proteobacteria	Phenotype	2	0.013	0.532	0.618

[3] *df*: degrees of freedom; SS: sum-of-squares; *F*-ratio: F-statistic

Supplementary Table 5. ANOVA results[4] for differentially-associated taxa across phenotypes

Strongylocentrotus purpuratus		Effect	*df*	SS	*F*-ratio	*p*
	Bradyrhizobiaceae	Phenotype	2	0.054	30.575	**0.001**
	Colwelliaceae	Phenotype	2	0.038	15.692	**0.004**
	Oleispira	Phenotype	2	0.073	17.583	**0.003**
	Pseudomonas	Phenotype	2	0.015	24.554	**0.001**
Mesocentrotus franciscanus		Effect	*df*	SS	*F*-ratio	*p*
	Colwelliaceae	Phenotype	2	0.003	15.373	**0.007**
	Sphingomonas	Phenotype	2	0.002	5.472	0.055
Strongylocentrotus droebachiensis		Effect	*df*	SS	*F*-ratio	*p*
	Flavobacteriaceae	Phenotype	2	0.020	1.596	0.291
	Bradyrhizobiaceae	Phenotype	2	0.004	1.260	0.360
	Polaribacter	Phenotype	2	0.020	1.753	0.265

[4] *df*: degrees of freedom; SS: sum-of-squares; *F*-ratio: F-statistic

Supplementary Note 1. Informatic pipeline for processing 16S rRNA datasets from MiSeq

Starting with raw read files from MiSeq:

1) Pair forward and reverse files using PEAR (pear-0.9.10-bin-64)
2) Trim paired files using Trimmomatic (trimmomatic-0.36.jar)
3) Convert paired and trimmed .fastq to .fasta using the custom code: cat [input .fastq] | paste - - - - | cut -f 1,2 | sed 's/^/>/'| tr "\t" "\n" >[output .fasta]
4) Validate tab-deliminted mapping file using "validate_mapping_file.py"
5) Generate meta-.fasta files using "add_qiime_labels.py"
6) Detect chimeras from meta-.fasta (called, "combined_seqs.fna")
7) Filter chimeras using "filter_fasta.py"
8) Pick OTUs using "pick_open_reference_otus.py"
9) Filter OTUs with >10 reads using "filter_otus_from_otu_table.py"
10) Filter 'o__Cryptophyta' using "filter_taxa_from_otu_table.py"
11) Determine rarefaction depth using "biom summarize-table"
12) Split filtered .biom table to test specific hypotheses using "split_otu_table.py"
13) Comparison of microbial communities via PCoA using "jackknifed_beta_diversity.py"
14) Statistical comparisons of rarefied matrices using "compare_categories.py"
15) Compare taxa from '13' using "summarize_taxa_through_plots.py"
