# Supplementary material for: Convergent shifts in host-associated microbial communities across environmentally elicited phenotypes
Source: Nat Commun. 2018 Mar 5;9:952. doi: 10.1038/s41467-018-03383-w (PMC5838112; doi:10.1038/s41467-018-03383-w)
Supplement: Supplementary file 3 — Description of Additional Supplementary Files [file 41467_2018_3383_MOESM3_ESM.pdf]

Description of Supplementary Files

File Name: Supplementary Data 1
Description: Cryptophyta sequences extracted from the biom table using 'biom convert' and 'Seq_Extract.py' in QIIME.

File Name: Supplementary Data 2
Description: BLAST (NCBI) results of the extracted Cryptophyta sequences.

File Name: Supplementary Data 3
Description: Sequences from BLAST result of the extracted Cryptophyta sequences.
